# Zebrafish as a Model for Anticancer Nanomedicine Studies

**DOI:** 10.3390/ph14070625

**Published:** 2021-06-28

**Authors:** Hissa F. Al-Thani, Samar Shurbaji, Huseyin C. Yalcin

**Affiliations:** 1 Biomedical Research Center, Qatar University, Doha P.O. Box 2713, Qatar; hissa.althani@qu.edu.qa (H.F.A.-T.); ss1104227@student.qu.edu.qa (S.S.); 2 Department of Biomedical Science, College of Health Sciences, QU Health, Qatar University, Doha P.O. Box 2713, Qatar

**Keywords:** zebrafish, cancer, nanomedicine, nanoparticle, pre-clinical

## Abstract

Nanomedicine is a new approach to fight against cancer by the development of anticancer nanoparticles (NPs) that are of high sensitivity, specificity, and targeting ability to detect cancer cells, such as the ability of Silica NPs in targeting epithelial cancer cells. However, these anticancer NPs require preclinical testing, and zebrafish is a useful animal model for preclinical studies of anticancer NPs. This model affords a large sample size, optical imaging, and easy genetic manipulation that aid in nanomedicine studies. This review summarizes the numerous advantages of the zebrafish animal model for such investigation, various techniques for inducing cancer in zebrafish, and discusses the methods to assess cancer development in the model and to test for the toxicity of the anticancer drugs and NPs. In addition, it summarizes the recent studies that used zebrafish as a model to test the efficacy of several different anticancer NPs in treating cancer.

## 1. Introduction

Nanoparticles (NPs) are defined as particles with a diameter between 1 and 100 nm or as fibers spanning the range of 1 to 100 nm according to the American Society of Testing and Materials (ASTM), International Organization for Standardization (ISO), and National Institute of Occupational Safety and Health (NIOSH) [1]. The NPs’ synthesis, analysis, and applications have grown exponentially over the last decade, making it an active area of powerful innovation [2]. The recent advances in industry and science allowed for the synthesis of nanoscale particles with several different properties known as multifunctional NPs that include carbon nanotubes (CNT), nano polymers, metal and metal oxides NPs, crystalline materials, and fullerenes [3,4]. Therefore, nanoparticles are one of the promising and favorable materials to be applied for many applications such as in treating cancers for targeted therapy with minimal damage to surrounding tissues. This is because of their special properties, their small size, and their high surface area to volume ratio that enhance surface interaction and cellular uptake [5].

NPs had a wide range of several different applications in many fields. For instance, in manufacturing, the particles are utilized as chemically inert additives, as fillers, pigments, or even anti-caking, but increasingly to make functional surfaces/membranes that possess catalytic, UV protection, anti-microbial, filtration, and other various properties [6]. Moreover, NPs can be utilized in medicine, a new discipline known as nanomedicine, that stands at the intersection of medicine, chemistry, and physics and focuses on biomedical purposes [7]. The applications include the utilization of NPs as biosensors for drugs, pathogens, nucleic acids, metabolites, proteins, and cancer cells by taking advantage of their electrochemical, photoluminescence, piezoelectric and optical properties [6,8]. In addition, those particles could be applied in drug delivery, bioimaging, tissue engineering, photoablation, and other therapeutic usages that could be facilitated by the accessibility of these small molecules through the body, conjugating with specific targeting modalities and in carrying functional load [4,8,9].

The current efficiency of anti-cancer drugs and quality of life in cancer patients during treatment has been improved due to the introduction of nanotechnology for cancer therapy [10]. There are over 25 approved nanomedicines by the Food and Drug Administration (FDA) and/or European Medicines Agency (EMA) and over 45 other NPs technologies, not FDA or EMA approved but are currently evaluated in ongoing trials that have been evolved over the past years [11]. The goal is to deliver the anti-cancer drugs that are loaded/ encapsulated inside the particles to increase the drug concentration at the targeted sites and at the same time reduces the off-target toxic effects [12]. Although the nano-therapies that are currently in use in the clinics, such as Vyxeos to treat myeloid leukemia and Onivyde to cure pancreatic cancer are capable to provide therapeutic efficacy similar to conventional cancer therapy, there is limited knowledge about the long-term toxicity caused by those particles, which suggests that nanomedicine discipline is still in its infancy [13].

Therefore, preclinical studies are necessary to improve the reproducibility of nanomaterials synthesis, assessment of the NPs‘ biological effects, therapeutic efficacy, and toxicity evaluation [14]. Over the past two decades, the zebrafish animal model became one of the popular models for studying human disease, especially for cancers [12] due to the ease in generating human tumors in this animal [15] and the late development of the adaptive immune system in the zebrafish, which is usually developed at 28 days post fertilization (dpf) [16]. Also, assessing NPs using this animal model is simpler than other animal models, because the fish embryos are optically transparent, and their tissues can be imaged in detail [17].

Despite all the advantages of this model, zebrafish has some limitations. For instance, there is a low number of the validated reagents and versatile tools that are suitable for use in zebrafish in contrast to the traditional mammalian models. This has limited the researchers from studying the details of the molecular and cellular mechanisms involved in cancer development. Moreover, zebrafish embryos need to be incubated at 28 °C, and this incubation temperature might not be suitable for the mammalian tumor cells’ metabolism, which requires 37 °C. Also, some nanoparticles such as water-insoluble NPs, could be toxic for the animal model leading to different types of toxicities and low survival rates [18].

The NPs are introduced into the zebrafish model through several different methods, mainly through exposing the animal directly to the particles found in the fish water [19,20] or through injecting the animal with the particles [21]. This review describes how to utilize zebrafish to study cancer nanomedicine. Also, this review shows the methods to assess cancer growth, anti-cancer drug efficacy, and the toxicities associated with the cancer treatments, as well as it provides an overview of studies using this model to study the efficacy and the toxicity of several NPs in treating cancers.

## 2. Zebrafish Cancer Models

Zebrafish (*Danio rerio*) is a common and prominent animal model in medical and biological research over the years. Zebrafish is a tropical freshwater fish of the *Cyprinidae* family under the Actinopterygii class and it mainly inhibits the Ganga river in the Indian subcontinent [22]. The first time the fish has been used as a research model was in the 1970s by George Streisinger because of its simplicity compared to the mouse model as well as its ease to be genetically manipulated. However, the use of zebrafish as an animal model increased in the 1990s as two scientists have used the model to develop two large mutant lines [23].

The zebrafish animal model has a lot of well-known characteristics and advantages that make it a good model for biological research and to study of human diseases [24]. The greatest advantages of this model are, the model is cost-efficient, rapid development, embryonic accessibility, optically transparent, genetic manipulability [25] and most importantly the physiological and genetic similarities with the humans including similar digestive tract [26], vascular system [27], brain [28] and immune system [29]. Also, about 70% of the human genes have functional similarities with zebrafish [30]. Furthermore, adult zebrafish are small in size allowing them to be cultivated in small freshwater tanks, thus less space required [30,31]. Also, researchers could use zebrafish at any development stage to conduct their experiments whether it’s in a larval stage (3 to 29 dpf) or the adult stage (90 dpf—2 years) [32].

Moreover, the zebrafish model has a lot of benefits for the clinical applications of nanoscale drug delivery systems, particularly, its optical transparency [33]. The transparency of the zebrafish embryo allows the researchers to dynamically observe inside the fish body such as organ or tumor development, vessel growth, and dispersion of nanomaterials [34]. The zebrafish embryos stay transparent until 60 h post-fertilization (hpf), at which stage the pigmentation process starts. To keep their transparency beyond that, the embryos are treated with 1-phenyl 2-thiourea (PTU), which inhibits the pigmentation process [35,36], or alternatively, the transgenetic models such as *Casper* can be used that lacks pigments on their skin [35]. In addition, due to the fast development of zebrafish embryos compared to other animal models, the screening time is less and the period for significant changes in the model morphology and behavior is shortened [37,38,39].

As zebrafish provides a great model for molecular studies for many human diseases, cancers are one of the most studied disorders using this model due to the simplicity of the model to be designed, through genetic engineering, xenoplantation, and chemical exposures [40,41,42,43]. For instance, overexpression of *Myc* gene would cause T-cell leukemia in zebrafish, exposing the fish to dimethylbenzanthracene would provoke intestinal cancer [44] and transplantation of human cancer cells such as the B16-F10 melanoma cells would develop melanoma in the model [45]. In addition, there are different approaches used to generate genetic, xenograft, and chemical models of zebrafish to investigate the alternations in molecular pathways and gene functions in cancer progression as well as to study novel anticarcinogenic drugs [46,47].

### 2.1. Xenograft Models

**Figure 1:**
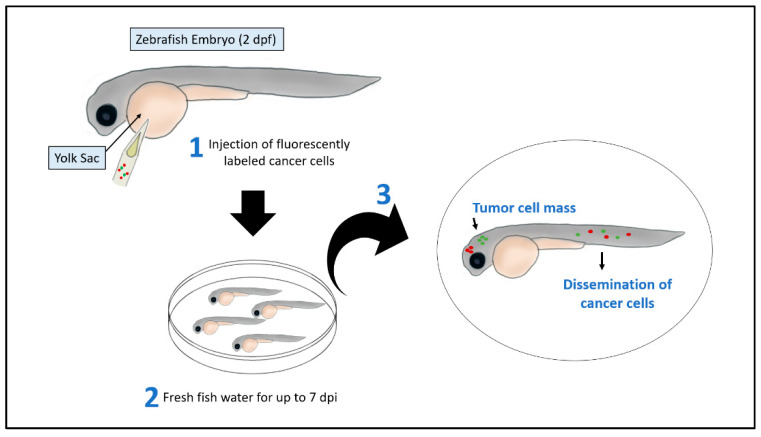
Xenotransplantation assay in zebrafish embryos. Human cancer cells stained with red- or green- fluorescent dye and injected alone or in combination using a glass micropipette or a glass capillary needle in the yolk sac of zebrafish embryos at 2 dpf. The xenografted embryos are then maintained at a specific temperature between 28 to 37 °C. After 2 to 7 days post-injection (dpi), the number of tumor cells increases, and cancer cells disseminate at distance sites such as head and tail.

Xenografted zebrafish embryos are one of the approaches for modeling cancer through transplanting human cancer cells into the animal model (Figure 1). The transplantation of cancer cells is possible at around 2 dpf when the zebrafish adaptive immune response is not yet established. Therefore, using 2 dpf embryos could allow the spread, metastasis, and survival of cancer cells without the need for a prior immunosuppressive treatment [16]. Several types of primary tumor cells or cancer cell lines have been transplanted to generate several different cancer xenograft zebrafish models such as, melanoma [48], leukemia [49], colorectal [50], kidney [51], breast [52], ovarian [53], prostate [54], pancreatic [55], oral [56] and lung [57] cancers’ models.

### 2.2. Genetic Models

Transgenic and mutant zebrafish models are major and powerful utilizing models for diseases and cancer research as these models are generated by different techniques, mainly through forward or reverse genetic approaches [24,58]. Forward genetic such as using ethylnitrosourea and reverse genetic by CRISPRs and TAL-like effector nuclease [58]. Also, high conservation of oncogenes such as *NRAS*^Q61K^ and *BRAFV*^600E^ in zebrafish cancer models and the microinjection of human tissue-specific promoters lead to the development of tumors with phenotypes analogous to human cancers [40].

The reverse genetic approaches through the specific knockdown or knockout genes of interest had given a rapid explanation of the functions of those genes. Furthermore, for two decades the only vertebrate model in which reverse genetics techniques have been done is the mouse model. These techniques’ protocols were based on the homologous recombination in the embryonic stem cells in vitro [59,60]. However, there are attempts to create embryonic stem cell lines of the zebrafish model but still, no protocol has been yet established for zebrafish for an embryonic stem cell-based knockout by homologous recombination [60].

Furthermore, another reverse genetic approach is the microinjection of the antisense morpholino oligonucleotides into the zebrafish embryos’ blastomeres or yolks in early stages [61,62,63]. This injection of a morpholino into a specific target gene would prevent the targeted gene translation [61,62,63].

**Figure 2:**
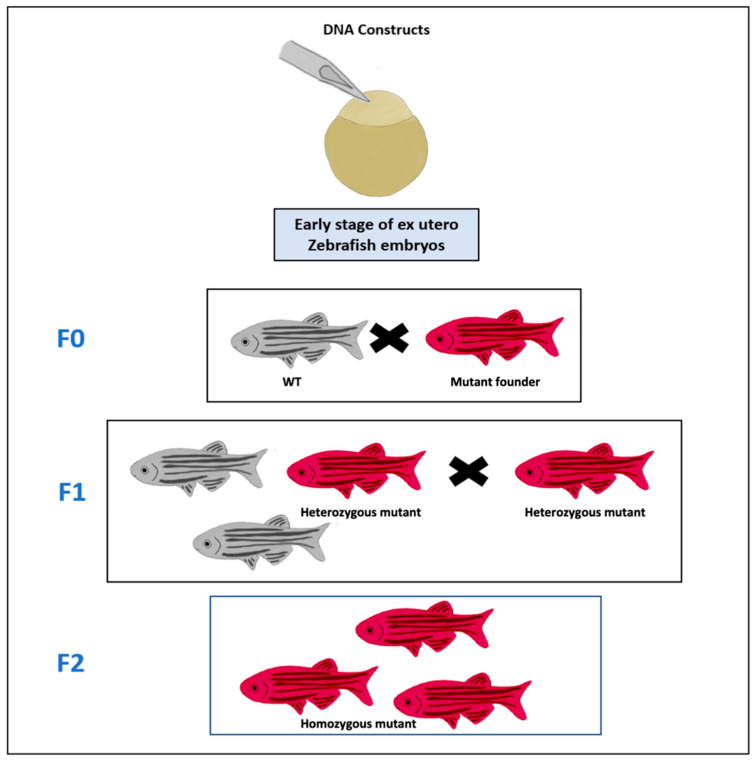
Transgenic cancer zebrafish lines. DNA constructs of cancer gene are microinjected into the cell of fertilized egg which is an early stage of zebrafish embryos. The adult mutant founder fish (Red) that have the germ cells, in which the construct was integrated into the genome, is mated with a wild-type (WT) zebrafish (Gray) at F0 to produce the F1 generation of fish, in which some of the progenies are heterozygous (Red) for the constructs and these heterozygous fish are then mated with each other to produce the F2 generation of fish that are homozygous to the construct (Red).

While the forward genetic screening or target-selected mutagenesis followed and random mutagenesis are other genetic approaches that are followed by genetic screening and mapping to look for specific genes’ functions, products, and mutations in the model [24,64]. Different techniques have been used for the target-selected mutagenesis in the zebrafish embryos’ genome such as the targeting induced local lesions in genomes (TILLING) using chemical mutagens like the *N*-ethyl-*N*-nitrosourea (ENU) [43,65,66]. As a result of those techniques many stable mutant zebrafish lines with mutations in a ribosomal protein (*RP*) or specific tumor suppressor genes such as *p53*, adenomatous polyposis coli (*APC*), and neurofibromatosis type 2 (*NF2*) genes, have been generated [43,64,65,67,68,69]. For example, a mutation in the wild-type p53 DNA-binding domain of the tp53^M214K^ mutant zebrafish model line could spontaneously develop the malignant peripheral nerve sheath tumors at eight and half months and 16.5 months with an incident of 28% [69]. Moreover, microinjection of DNA constructs in early zebrafish embryos’ stage (Figure 2) with various tissue-specific promoters and systems to accomplish the spatial and temporal control of the expression of some transgenes such as the GAL4-*UAS* and Cre-*LoxP* and *Tol2* transposon and LexPR binary systems had led to the development of several transgenic zebrafish cancer models [24,70,71,72].

### 2.3. Chemical Models

**Figure 3:**
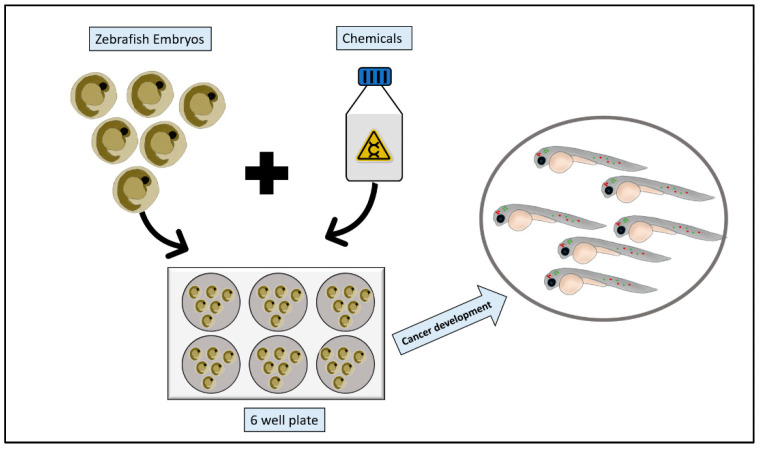
Zebrafish chemical carcinogenesis model. Zebrafish embryos are collected, and healthy embryos are arrayed into a multi-well plate. Chemical carcinogens are then added to the multi-well plate following dissolving or suspending them in the fish water. After appropriate incubation period, embryos are screened for cancer growth.

The use of chemical carcinogens was one of the first approaches to induce cancers’ mutations for tumor formation or developmental defects in the zebrafish animal model by which the animal is exposed to the carcinogenic chemicals that are dissolved or suspended in the fish’s swimming water thus allowing for long term exposure to those chemicals [58] (Figure 3). The Zebrafish animal model is the most responsive model to the different carcinogens as indicated by some studies and this animal model had also shown a greater diversity of neoplasm mutations and types than other kinds of fish species [73].

For example, exposing the zebrafish embryos to *N*-methyl-*N*-nitro-*N*-nitrosoguanidine (MNNG) and dimethylbenzanthracene (DMBA) had shown to induce a large number of different tumors in the animal, such as hemangiomas, rhabdomyosarcomas, leiomyosarcoma, chondromas, seminomas, hemangiosarcomas, and most commonly the hepatic neoplasms [73,74,75]. Moreover, the *N*-nitrosodiethyl- amine (DEN) showed to induce pancreatic and liver carcinomas, while the *N*-nitrosodimethylamine (NDMA) mainly induces liver cancers [76,77]. Also, some of these chemical carcinogens when applied to mutant fish with a cancer genetic disposition, these carcinogens treatment would increase the fish sensitivity to induce tumors compared to the treated wild-type fish [65,78]. And the advantage of the chemical carcinogens approach is that specific tumors are easy to study as they could be induced effortlessly [58].

## 3. Tumor Growth and Metastasis Assessment

Currently, there is a demand for utilizing new techniques for visualizing the spread and disruption of the tumor microenvironment, for drug testing and responses to treatments in the animal models [79]. Thus, the zebrafish animal model is one of the optimum animal models to study tumors, because tumor engraftment and growth are very rapid and could provide readouts within days compared to other models [41,80]. Moreover, xenografted zebrafish could absorb small molecular weight compounds directly from the surrounding water, making them a good model to test for anti-cancer drugs [81].

Several approaches have been developed to quantify and assess the human tumor cells’ proliferation that is xenografted in the zebrafish embryos [79]. For instance, with the aid of a fluorescent microscope and image analyzer’s software, the fluorescently-labeled cancer cells could be quantified in the embryos over a time interval from the day of injection, which is usually at 48 dpf or 72 dpf until the end-point day (usually around 7dpf) [82,83]. Another approach to determine tumor cells proliferation is by enzymatically dissociate the embryos at the endpoint time into a single cell suspension and then the number of fluorescent cells is counted in the suspension and this number is divided by the 24h cells number to show the fold increase in cell number [48,84] or through a flow cytometry after enzymatically digesting the embryos [79].

To determine if the cancer cells have been disseminated through the zebrafish embryo’s body, the cells would be labeled by a fluorescent protein or stained by a chemical dyestuff [85] and the cancer cells’ metastasis would be imaged inside the embryo body within two days after the injection [86] and these fish could then be imaged using confocal microscopy and analyzed through software such as Fiji, so the number of the metastatic cells is determined [87]. For instance, Augustine and his colleagues have used a green fluorescent protein (GFP) expressing cancer cells to detect and quantify migrated cancer cells in ovo [88]. Moreover, to evaluate tumor progression, qPCR could be used to detect a cancer-specific gene or a human housekeeping gene [54,89]. In addition, Gotra et al., has studied human cancer dissemination through the xenograft model by a quantitative bioimaging platform [90]. While Benjamin and Hynes have used a transparent adult zebrafish with a homozygous mutation in two pigmentation loci, called *Casper*, that has been described by White et al. [35]. This mutant zebrafish allowed visualizing the in vivo metastasis [91].

## 4. Anti-Cancer Efficacy Assessment

To evaluate rapidly the therapeutic efficacy of some molecular compounds, the zebrafish embryos of the wild-type, mutant or transgenic lines would be incubated along with the compounds in water and the use of microplates could facilitate the comparison quantification analysis of the incubated embryos, as this technique is similar to the cell-based assays [92]. Moreover, the use of fluorescent reporter molecules with several transgenic lines would aid in a further detailed examination of functional and morphological changes occurring in the model [93]. And due to the high number of established cancer xenografted zebrafish models, the researchers could demonstrate the efficacy of multiple molecules and compounds to treat cancer, by exposing the xenografted embryos with the compound or by treating the cancer cells with the compounds before implanting them into the embryos [94,95]. Adult zebrafish could also be utilized to assess the efficacy of anti-cancer agents, as the tumors are disseminating through the adult fish from 5 to 10 days after transplantation and the definite appearance of the tumor cells would occur 2 to 3 weeks post the transplantation [35].

To illustrate how the xenograft and transgenic or mutant zebrafish model have given advantages to the researchers to establish candidate compounds to treat cancers, Ridges et al. have shown that the Lenaldekar compound was able to eradicate immature T-cells, which have similar development with the malignant T-cells, without affecting the cell cycle of other cells type [96] by using a genetically engineered T-cell reporting zebrafish larvae (*lck:EGFP*) [97]. Moreover, Pruvot et al. had validated the anti-cancer efficacy of Oxaphorines and Imatinib with the aid of a xenografted zebrafish model injected with human leukemia cell lines [49].

### 4.1. Toxicity Assessment

The outburst of the nanotechnology, chemical engineering, pharmaceutical industry, and other industries that manufacture consumer and therapeutic products had overtaken the toxicity assessments of these chemical products [98]. Therefore, there is a large demand for high-throughput biological model systems to test for the toxicity of the chemicals [99], many several models are being tested and utilized for this demand over the past two decades, the zebrafish, which are complex vertebrates, had been determined as a complementary animal model to the mammals for toxicity assessments [100].

This is due to zebrafish’s highly conserved organ systems and metabolic pathways that could be used to evaluate several toxicological outcomes and extends the acute toxicity into much detailed mechanistic studies [101]. As the NPs engineering industry is producing a wide range of nanomaterials [102], the main purposes and application of the NPs are in the electronics and medicine fields [103,104]. Therefore, toxicological analysis of those particles is very significant to validate their usage [105].

The in vivo toxicity analysis has been done traditionally on rat, mouse, frog, and other animal models, however due to their many difficulties such as their maintenance, growth, ethical approval, and the scarifications [106,107]. Thus, the alternative model is the zebrafish animal which is much simpler to be maintained and they possess analogs organs and tissues with similar function and structures as the human, including the brain, heart, kidney, and liver that made it easier to determine the toxicity of the particles on those tissues and organs [108].

### 4.2. Behavioral and Neurotoxicity

**Figure 4:**
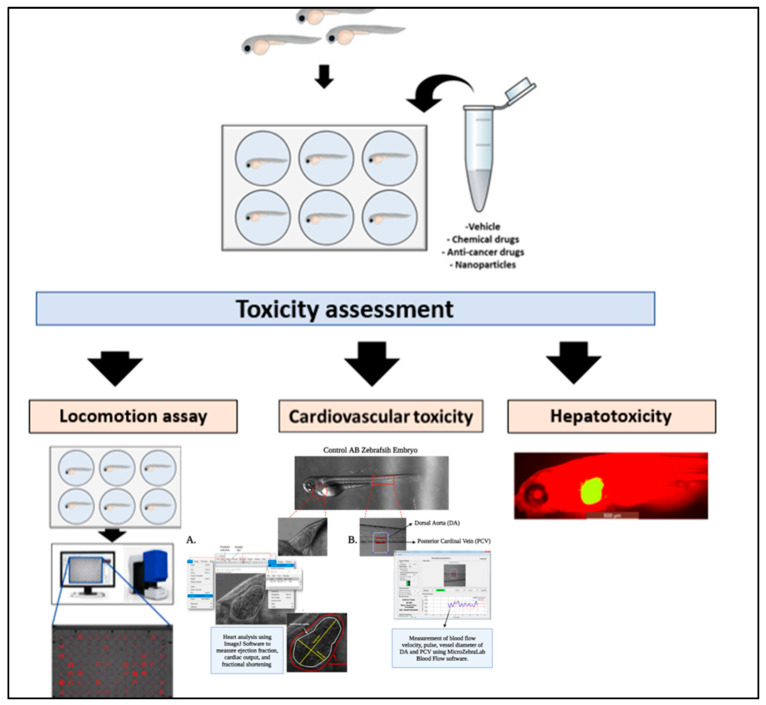
Toxicity assessments. Wild-type, mutant, transgenic, or xenografted zebrafish embryos are exposed to the testing vehicle e.g., chemicals, anticancer drugs, or NPs in a multi-well plate. At the end of the incubation period, toxicity analysis of zebrafish systems can be performed to determine the therapeutic effects of the tested materials. For example, behavioral and neurotoxicity can be assessed by studying locomotion, spatial recognition, and color preference as well for zebrafish’s brain development. Cardiovascular toxicity can be assessed through the change in the heart shape, rhythm, blood flow velocity and expression of cardiac failure gene markers. While hepatotoxicity can be determined by the development of hepatic hypoplasia.

A range of complex behaviors has been used as perceptive parameters to assess the toxicity in the zebrafish such as locomotion, spatial recognition, and color preference. [109,110]. The nervous system that supports these parameters and behaviors plus the brain development, which predominantly vulnerable to oxidative stress due to its high energy demand, the cellular content of lipids and proteins, and low levels of antioxidants [7]. In addition, after 5 days of fertilization, the zebrafish juveniles could swim freely, and their brain tissues would be already differentiated into the structures of the diencephalon, hindbrain, telencephalon, midbrain, and ganglia that could also be used to assess the behavioral toxicity like on the animal memory, motion, and learning. Also, neurotoxicity could be evaluated by the morphological changes, biomedical indicators, apoptosis, and necrosis of the zebrafish neurons [111] (Figure 4).

Several NPs could trigger free radical activity at their surfaces that create oxidative stress at the site of particle deposition and translocation [112,113] and neurotoxicity has been noticed in case if the particles were able to reach the brain which they can also lead to neurodegeneration [114,115]. Moreover, some particular NPs can affect animal behavior, for example, silicon dioxide (siO_2_) NPs were found to cause altered color preferences [116], cadmium telluride (CdTe) quantum dots could affect the locomotor activity [117], and titanium dioxide (TiO_2_) NPs exposure could alter gene expression and enhance neuron apoptosis and glial cell proliferation [118].

Pang et al. have also tested for the neurotoxicity of a number of drugs, such as Ethanol, Taxol, Acrylamide and 6-hydroxydopamine (6-OHDA). They have looked for the pharmacological effects of those drugs on the motor and dopaminergic neurons and on the optic nerves. This was done by visualizing several neuronal cell types and through performing whole mount immunostaining. Also, to sasses the neurotoxicity of those drugs on the zebrafish, they have used TUNEL staining, in situ hybridization and immunostaining in order to assess the neuronal proliferation, apoptosis, oxidation as well as the integrity of the myelin sheath [119].

Furthermore, the zebrafish larvae have been exposed to two different sizes (10 and 50 nm) of the polyvinylpyrrolidone-coated AgNPs (AgNP-PVP) and their swimming behavior have been evaluated after 24 h of exposure by determining their responses to different lighting conditions, the larvae have showed decreased locomotor activity with the 10 nm AgNP-PVP, whereas the 50 nm AgNP-PVP caused hyperactivity [120]. While de Oliveria et al. had exposed the animals to 200 mg/kg dextran-coated Fe_2_O_3_NPs and tested them after 24 h, the results showed a reduction in the acetylcholinesterase activity, decrease in the exploratory performance, a significant increase of ferric iron levels in the brains, and *casp8*, *casp9*, and *jun* genes induction, these findings are suggesting brain toxicity by the induction of apoptosis and inhibition of acetylcholinesterase [121].

### 4.3. Cardiovascular Toxicity

Zebrafish embryos are a suitable model to assess cardiotoxicity of various compounds such as, NPs, as the embryos hearts resemble the human embryonic heart with one ventricle, one atrium, and valves between the atrioventricular compartments with a regular heartbeat that starts after 36 hpf [122]. Therefore, the microscope could be used to look for the heart shape and rhythm such as the heartbeats, blood vessels’ cell activity, and morphology [111] (Figure 4). To assess zebrafish cardiac function and blood flow hemodynamics, Benslimane and her colleagues have established high-speed microscopy with a combination of an analysis software protocol for practical image acquisition for studying zebrafish’s cardiovascular system. They have provided a detailed calculation of several cardiac parameters and expected ranges for these cardiac parameters [123,124]. Moreover, Benslimane et al., have adapted a single-mode mice Doppler echocardiography system to assess the cardiac function of normal or diseased zebrafish and to measure the flow velocities of adult zebrafish [125].

Furthermore, Da’as et al., have used fluorescence microscopy imaging to assess the total and a ventricular number of cardiomyocytes in response to some zebrafish morphants. They have labeled the cardiomyocytes with GFP as well as labeling cardiac muscle cell plasma membrane with ZN-8 antibody along with 4′,6-diamidino-2-phenylindole (DAPI) stain. The zebrafish whole heart is then imaged under fluorescence microscopy by scanning it anteriorly to posteriorly [126]. Also, complicated blood flow patterns during the development of the zebrafish cardiovascular system could be tracked and analyzed using Digital particle image velocimetry (DPIV) and computational fluid dynamics (CFD) modeling [127,128].

The successful establishment of transgenic zebrafish lines had aided in the monitoring, quantifying, and qualifying cardiovascular damage caused by certain NPs. For instance, the effects of CuONPs exposure on the vasculogenesis or angiogenesis were determined using the zebrafish transgenic *Tg* (nacre/fli1:EGFP) line and it has been found that CuONPs inhibit vasculogenesis through the reduction of the vascular endothelial growth factor (VEGF) expression and the initiation of apoptosis [129]. Moreover, the toxic effects on hematopoiesis by particular NPs were studied. For example, the toxic effects of AgNPs were assessed through the transcriptional responses of zebrafish embryos at 24 hpf, using microarray analysis. The AgNPs had downregulated hemoglobin genes as indicated by the gene ontology analysis and this reduction was further proved using *O*-dianisidine staining, whole-mount in situ hybridization, and quantitative reverse transcription-polymerase chain reaction [130].

### 4.4. Hepatotoxicity

Zebrafish’s livers in the early developmental stages could respond to chemicals similar to the human liver, thus it is considered an ideal model to assess hepatotoxicity of the NPs for instance [111]. Villacis et al. had investigated the acute toxicity of IONPs in adult zebrafish after exposing the particles to the fish for 96 h, using different endpoints. The highest concentrations of the particles (37.2 and 74.4 mg/L) had caused oxidative stress in the liver cells [131].

In addition, high doses of CuONPs can cause hepatic hypoplasia when exposed to zebrafish embryos and larvae for a short time [132]. ZnONPs had also been shown to cause hepatotoxicity in the zebrafish due to oxidative damage, which was determined by the high levels of malondialdehyde, a biomarker of the oxidative effects [133] (Figure 4).

## 5. Anti-Cancer NPs Tested on Zebrafish

Developing NPs that can identify, target, and eliminate cancer cells is a great aim in the field of nanomedicine, however, to test those particles, an ideal animal model should be selected to determine the efficacy and toxicity of the NPs [134]. Although rodent models are the widely used preclinical animal model, their high cost, the long time required to generate cancer models, and due to some visibility issues, such as monitoring tumor cell migration is challenging in this animal model [135,136]. Therefore zebrafish have been designated as an alternative model to test those particles, due to the several advantages that have been stated previously.

Consequently, several NPs have been developed that are conjugates with the targeting ligands and/or loaded with the chemotherapeutic drugs, to treat cancers through testing their efficacy on zebrafish [137,138].

### 5.1. Gold and Platinum NPs

Gold (Au) and platinum (PT) NPs are ideal candidates for cancer therapy, as Au NPs can generate heat in the presence of infrared radiation [139] and PT NPs could release PT ions by surface oxidation that provides the anti-cancerous property [140]. To determine the toxicity effects of these particles on the zebrafish, Asharani et al. found that Pt-NPs had caused delays in the hatching rate and drop in heart rate, axis curvatures, and touch response, while the Au-NPs showed no indication of toxicity [141]. Despite the advantages of Au and Pt-NPs in treating cancers, no previous studies have been done on the zebrafish cancer model using those NPs (Table 1).

### 5.2. Dendrimers NPs

Dendrimers are an emerging type of nanoscale, monodisperse polymers that are applied in a wide range of industries such as in medicine to be used as delivery systems for gene therapy and targeted drug delivery for tumor cells or as dendritic sensors [142]. Bodewein et al. had exposed the polyamidoamine (PAMAM) dendrimers of generations G3.0, 3.5, 4.0, 4.5, and 5.0 and polypropylenimine (PPI) dendrimers (G3.0, 4.0 and 5.0) to the zebrafish embryos for 96 h to assess the toxicity of those particles. The results showed that the toxicity of cationic PAMAM and PPI dendrimers had increased over time and the predominant effects were mortality and a reduction in the heartbeats and blood circulation for the PPI dendrimers. However, for the anionic PAMAM of generation G3.5 and G4.5, there was no toxicity determined [143]. To test for the efficacy of dendrimers NPs against cancer cells, Wu et al., had shown that PAMAM-conjugated histidine and cysteine (GH and GHC) NPs loaded with the chemotherapeutic drug, doxorubicin (DOX) inhibited the proliferation of the cervical cancer cells, in zebrafish embryos [144] (Table 1).

### 5.3. Polymersome NPs

Kocere et al. had injected mouse melanoma B16 cells into zebrafish’s neural tube at 3 dpf to generate a melanoma xenograft zebrafish model to test for polymersome NPs (PEG-PDPA NPs) loaded with doxorubicin on this model. The PEG-PDPA NPs were injected intravenously at 4 dpf. The particles had been selectively accumulated at the neural tube cancer region and on some individual cancer cells and tumor-associated macrophages. And when doxorubicin was released from the particles a reduction in the systemic toxicity was seen compared to the toxicity of the free drug alone and a reduction in the cancer cell signal (less fluorescence signal) was seen after seven days of injection of the particles to the xenografted zebrafish [145] (Table 1).

### 5.4. Porphyrin-Based Bridged Silsesquioxane NPs

A new class of NPs known as bridged silsesquioxane NPs (BSNs), is currently exhibiting versatile applications and acts as a strong potential for nanomedicine [146]. Dib et al. had applied these particles to treat and target breast tumors in vivo, by synthesizing BNSs from an octasilylated functional porphyrin precursor (PORBSNs). Also, the particles were conjugated with PEG and mannose (PORBSNs-mannose) to target the breast cancer cells. Then the particles were intravenously injected into the 4 dpf zebrafish model that bearing the human breast tumor. After one and half hours the tumor areas were excited with a two-photon beam-induced focused laser and this results in a strong reduction in the tumor size and activation of apoptosis pathways [147]. No toxicity has been seen on the xenografted zebrafish embryos after NPs injection [147] (Table 1).

### 5.5. Hydroxyapatite NPs

Nadar et al. have evaluated the ability of novel hydroxyapatite (HA) NPs loaded with a kiteplatin-pyrophosphate compound, which is PtPP ([Pt (dihydrogenpyrophosphate)(cis-1,4-DACH)]) to reduce the survival of breast cancer cells [148]. The toxicity of the HA NPs alone has been studied by Xu et al. who showed that these particles could aggregate into bigger particles around the membrane proteins as well as cause little developmental issues after exposing them to 2 hpf zebrafish embryos [149]. However, in the Nadar et al. study, they have injected these particles directly into the blood circulation of the 2 dpf zebrafish embryos through the duct of Cuvier and no toxicity or phenotypic abnormalities have been developed. Thus, they have shown a reduction in cancer cell survival after co-injection breast cancer cells with citrate-functionalized and the PtPP-loaded HA NPs [148] (Table 1).

**Table 1:** Anticancer NPs and their anticancer efficacy and associated toxicity on zebrafish model.

Anticancer NPs	Cancer Type:	Outcomes:	Toxicity:	References
Au and PT NPs	No previous studies		PT-NPs: - Delays in hatching rate - Low heart rate, axis curvatures & touch response Au-NPs - No indication of toxicity	[141]
Dendrimers NPs	Cervical Cancer	Inhibition of cancer cells proliferation	PPI dendrimers: - Mortality - Reduction in heartbeats & blood circulation	[143,144]
Polymersome NPs	Melanoma	Reduction in cancer cells	- No indication of toxicity	[145]
Porphyrin-based bridged silsesquioxane NPs	Breast Cancer	Reduction in tumor size & activation of apoptosis pathways	- No indication of toxicity	[147]
Hydroxyapatite NPs	Breast Cancer	Reduction in cancer cells’ survival	HA NPs: - Aggregates around membrane proteins - little toxicity to development	[148,149]
Silica NPs	Epithelial Cancers	Fast-targeting capability of epithelial tumors	Silica NPs: - Decrease zebrafish embryos’ hatching rate - Increase mortality and cell death - Embryonic malformation	[150,151]

### 5.6. Silica NPs

Ultrabright fluorescent silica NPs had shown a fast-targeting capability of the epithelial tumors in vivo [150]. Peerzade et al. demonstrated the targeting ability of the particles on a zebrafish animal model transplanted with human cervical epithelial tumors (HeLa cells). They found that when folate molecules are added to the particles, the particles become more specific to the epithelial cancers, in which it has targeted the tumor cells as small as 10–20 microns within 20–30 min after they injected them into the rich capillary bed located behind the 3 dpf animal eye to enable the particles to enter the circulation. The toxicity of the particles was studied due to the presence of surfactant, the toxicity study was done using keratinocyte cells of normal human skin. The toxicity of the particles showed to be about 22 times less toxic compared to Triton X-100 surfactant. Also, the final concentration of the particles that were injected into the animal blood showed to be 10 times smaller than the CC_50_ toxicity concentration found in the normal cells [150]. However, a study performed by Duan et al. revealed that high exposure dosages of SiNPs to 4–96 hpf embryos, cause a decrease in zebrafish embryos’ hatching rate and an increase in mortality and cell death [151]. Also, exposure to SiNPs has caused embryonic malformation such as yolk sac and pericardial edema and head and tail malformations [151] (Table 1).

## 6. Conclusions

The Zebrafish animal model is a good animal model for developing and testing anticancer NPs as this model provides several advantages over other types of laboratory animals. Zebrafish are cost-effective, resembles a high homology to humans, rapidly develop, optically transparent, and easy to be genetically manipulated. The various advantages made the generation of human cancers in the zebrafish more easily either by injection of the human cancer cells, chemically introduce cancerous mutations, or through genetic engineering, and these cancer models helped in studying cancer development and prognosis in response to the anticancer nanotherapeutics. Moreover, to test for the efficacy of those NPs, different methods have been created to test for the cancer development and metastasis in zebrafish such as detecting the fluorescently labeled cancer cells in the animal body. Also, due to the importance of toxicity screening of any new therapy, zebrafish afford a rapid NPs toxicity screening at the cardiovascular, hepatic level and on the neurological and behavioral level. Lastly, many recent studies with zebrafish models have been performed to test for different types of anticancer NPs in treating cancers such as gold, platinum, dendrimers, polymersome, porphyrin-based bridged silsesquioxane, hydroxyapatite, and silica NPs, and most of them showed high ability in treating cancer.

## Data Availability

Not applicable.

## References

[1] Horikoshi S., Serpone N. (2013). Microwaves in Nanoparticle Synthesis: Fundamentals and Applications.

[2] De Crozals G., Bonnet R., Farre C., Chaix C. (2016). Nanoparticles with multiple properties for biomedical applications: A strategic guide. Nano Today.

[3] Seaton A., Tran L., Aitken R., Donaldson K. (2009). Nanoparticles, human health hazard and regulation. J. R. Soc. Interface.

[4] McNamara K., Tofail S.A. (2013). Nanoalloys: 10. Biomedical Applications of Nanoalloys.

[5] Shurbaji S., Anlar G.G., Hussein E.A., Elzatahry A., Yalcin H.C. (2020). Effect of Flow-Induced Shear Stress in Nanomaterial Uptake by Cells: Focus on Targeted Anti-Cancer Therapy. Cancers.

[6] Stark W.J., Stoessel P.R., Wohlleben W., Hafner A. (2015). Industrial applications of nanoparticles. Chem. Soc. Rev..

[7] Haque E., Ward A.C. (2018). Zebrafish as a Model to Evaluate Nanoparticle Toxicity. Nanomaterials.

[8] Das S., Mitra S., Khurana S.M.P., Debnath N. (2013). Nanomaterials for biomedical applications. Front. Life Sci..

[9] McNamara K., Tofail S.A.M. (2015). Nanosystems: The use of nanoalloys, metallic, bimetallic, and magnetic nanoparticles in biomedical applications. Phys. Chem. Chem. Phys..

[10] Sun T., Zhang Y.S., Pang B., Hyun D.C., Yang M., Xia Y. (2014). Engineered Nanoparticles for Drug Delivery in Cancer Therapy. Angew. Chem. Int. Ed..

[11] Anselmo A.C., Mitragotri S. (2019). Nanoparticles in the clinic: An update. Bioeng. Transl. Med..

[12] Evensen L., Johansen P.L., Koster G., Zhu K., Herfindal L., Speth M., Fenaroli F., Hildahl J., Bagherifam S., Tulotta C. (2015). Zebrafish as a model system for characterization of nanoparticles against cancer. Nanoscale.

[13] Foulkes R., Man E., Thind J., Yeung S., Joy A., Hoskins C. (2020). The regulation of nanomaterials and nanomedicines for clinical application: Current and future perspectives. Biomater. Sci..

[14] Ioannidis J.P.A., Kim B.Y.S., Trounson A. (2018). How to design preclinical studies in nanomedicine and cell therapy to maximize the prospects of clinical translation. Nat. Biomed. Eng..

[15] Amatruda J.F., Shepard J.L., Stern H.M., I Zon L. (2002). Zebrafish as a cancer model system. Cancer Cell.

[16] Lam S., Chua H., Gong Z., Lam T., Sin Y. (2004). Development and maturation of the immune system in zebrafish, Danio rerio: A gene expression profiling, in situ hybridization and immunological study. Dev. Comp. Immunol..

[17] He S., Lamers G.E., Beenakker J.M., Cui C., Ghotra V.P., Danen E., Meijer A.H., Spaink H., Snaar-Jagalska B.E. (2012). Neutrophil-mediated experimental metastasis is enhanced by VEGFR inhibition in a zebrafish xenograft model. J. Pathol..

[18] Zhao S., Huang J., Ye J. (2015). A fresh look at zebrafish from the perspective of cancer research. J. Exp. Clin. Cancer Res..

[19] Pitt J.A., Kozal J.S., Jayasundara N., Massarsky A., Trevisan R., Geitner N., Wiesner M., Levin E.D., Di Giulio R.T. (2018). Uptake, tissue distribution, and toxicity of polystyrene nanoparticles in developing zebrafish (*Danio rerio*). Aquat. Toxicol..

[20] Jurewicz A., Ilyas S., Uppal J.K., Ivandic I., Korsching S., Mathur S. (2020). Evaluation of Magnetite Nanoparticle-Based Toxicity on Embryo–Larvae Stages of Zebrafish (*Danio rerio*). ACS Appl. Nano Mater..

[21] Dal N.K., Kocere A., Wohlmann J., Van Herck S., Bauer T.A., Resseguier J., Bagherifam S., Hyldmo H., Barz M., De Geest B.G. (2020). Zebrafish Embryos Allow Prediction of Nanoparticle Circulation Times in Mice and Facilitate Quantification of Nanoparticle–Cell Interactions. Small.

[22] Dahm R., Geisler R. (2006). Learning from Small Fry: The Zebrafish as a Genetic Model Organism for Aquaculture Fish Species. Mar. Biotechnol..

[23] Khan F.R., Alhewairini S.S. (2019). Zebrafish (*Danio rerio*) as a Model Organism. Current Trends in Cancer Management.

[24] Mimeault M., Batra S.K. (2013). Emergence of zebrafish models in oncology for validating novel anticancer drug targets and nanomaterials. Drug Discov. Today.

[25] Berger J., Currie P. (2007). The role of zebrafish in chemical genetics. Curr. Med. Chem..

[26] Kanungo J., Cuevas E., Ali S., Paule M. (2014). Zebrafish Model in Drug Safety Assessment. Curr. Pharm. Des..

[27] Gore A., Monzo K., Cha Y.R., Pan W., Weinstein B.M. (2012). Vascular Development in the Zebrafish. Cold Spring Harb. Perspect. Med..

[28] Kalueff A.V., Stewart A.M., Gerlai R. (2014). Zebrafish as an emerging model for studying complex brain disorders. Trends Pharmacol. Sci..

[29] Lieschke G.J. (2001). Morphologic and functional characterization of granulocytes and macrophages in embryonic and adult zebrafish. Blood.

[30] Santoriello C., Zon L.I. (2012). Hooked! Modeling human disease in zebrafish. J. Clin. Investig..

[31] Nusslein-Volhard C., Dahm R. (2002). Zebrafish.

[32] Ruzicka L., Howe D.G., Ramachandran S., Toro S., E Van Slyke C., Bradford Y.M., Eagle A., Fashena D., Frazer K., Kalita P. (2019). The Zebrafish Information Network: New support for non-coding genes, richer Gene Ontology annotations and the Alliance of Genome Resources. Nucleic Acids Res..

[33] Chakraborty C., Sharma A.R., Sharma G., Lee S.-S. (2016). Zebrafish: A complete animal model to enumerate the nanoparticle toxicity. J. Nanobiotechnol..

[34] Lee K.Y. (2017). Zebrafish models for functional and toxicological screening of nanoscale drug delivery systems: Promoting preclinical applications. Biosci. Rep..

[35] White R.M., Sessa A., Burke C., Bowman T., LeBlanc J., Ceol C., Bourque C., Dovey M., Goessling W., Burns C.E. (2008). Transparent Adult Zebrafish as a Tool for In Vivo Transplantation Analysis. Cell Stem Cell.

[36] McGrath P., Li C.-Q. (2008). Zebrafish: A predictive model for assessing drug-induced toxicity. Drug Discov. Today.

[37] Lawrence C. (2007). The husbandry of zebrafish (*Danio rerio*): A review. Aquaculture.

[38] Berghmans S., Jette C., Langenau D., Hsu K., Stewart R., Look T., Kanki J.P. (2005). Making waves in cancer research: New models in the zebrafish. Biotechniques.

[39] Briggs J.P. (2002). The zebrafish: A new model organism for integrative physiology. Am. J. Physiol. Regul. Integr. Comp. Physiol..

[40] White R., Rose K., Zon L. (2013). Zebrafish cancer: The state of the art and the path forward. Nat. Rev. Cancer.

[41] Konantz M., Balci T., Hartwig U.F., Dellaire G., André M.C., Berman J.N., Lengerke C. (2012). Zebrafish xenografts as a tool for in vivo studies on human cancer. Ann. N. Y. Acad. Sci..

[42] Huang H., Vogel S.S., Liu N., A Melton D., Lin S. (2001). Analysis of pancreatic development in living transgenic zebrafish embryos. Mol. Cell. Endocrinol..

[43] Feitsma H., Cuppen E. (2008). Zebrafish as a Cancer Model. Mol. Cancer Res..

[44] Muth-Köhne E., Sonnack L., Schlich K., Hischen F., Baumgartner W., Hund-Rinke K., Schäfers C., Fenske M. (2013). The toxicity of silver nanoparticles to zebrafish embryos increases through sewage treatment processes. Ecotoxicology.

[45] Harfouche R., Basu S., Soni S., Hentschel D.M., Mashelkar R.A., Sengupta S. (2009). Nanoparticle-mediated targeting of phosphatidylinositol-3-kinase signaling inhibits angiogenesis. Angiogenesis.

[46] Bill B.R., Petzold A., Clark K.J., Schimmenti L., Ekker S.C. (2009). A Primer for Morpholino Use in Zebrafish. Zebrafish.

[47] Drabsch Y., Snaar-Jagalska B.E., Dijke P.T. (2016). Fish tales: The use of zebrafish xenograft human cancer cell models. Histology Histopathology.

[48] Haldi M., Ton C., Seng W.L., McGrath P. (2006). Human melanoma cells transplanted into zebrafish proliferate, migrate, produce melanin, form masses and stimulate angiogenesis in zebrafish. Angiogenesis.

[49] Pruvot B., Jacquel A., Droin N., Auberger P., Bouscary D., Tamburini J., Muller M., Fontenay M., Chluba J., Solary E. (2011). Leukemic cell xenograft in zebrafish embryo for investigating drug efficacy. Haematologica.

[50] Varas M.A., Muñoz-Montecinos C., Kallens V., Simon V., Allende M.L., Marcoleta A.E., Lagos R. (2020). Exploiting Zebrafish Xenografts for Testing the in vivo Antitumorigenic Activity of Microcin E492 against Human Colorectal Cancer Cells. Front. Microbiol..

[51] Wertman J., Veinotte C.J., Dellaire G., Berman J.N. (2016). The Zebrafish Xenograft Platform: Evolution of a Novel Cancer Model and Preclinical Screening Tool. Advances in Experimental Medicine and Biology.

[52] Drabsch Y., He S., Zhang L., Snaar-Jagalska B.E., Dijke P.T. (2013). Transforming growth factor-β signalling controls human breast cancer metastasis in a zebrafish xenograft model. Breast Cancer Res..

[53] Latifi A., Abubaker K., Castrechini N., Ward A.C., Liongue C., Dobill F., Kumar J., Thompson E.W., Quinn M.A., Findlay J.K. (2011). Cisplatin treatment of primary and metastatic epithelial ovarian carcinomas generates residual cells with mesenchymal stem cell-like profile. J. Cell. Biochem..

[54] Xu W., Foster B.A., Richards M., Bondioli K.R., Shah G., Green C.C. (2017). Characterization of prostate cancer cell progression in zebrafish xenograft model. Int. J. Oncol..

[55] Weiss F.U., Marques I.J., Woltering J., Vlecken D.H., Aghdassi A., Partecke L.I., Heidecke C., Lerch M.M., Bagowski C.P. (2009). Retinoic Acid Receptor Antagonists Inhibit miR-10a Expression and Block Metastatic Behavior of Pancreatic Cancer. Gastroenterol..

[56] Yu C.-I., Chen C.-Y., Liu W., Chang P.-C., Huang C.-W., Han K.-F., Lin I.-P., Lin M.-Y., Lee C.-H. (2018). Sandensolide Induces Oxidative Stress-Mediated Apoptosis in Oral Cancer Cells and in Zebrafish Xenograft Model. Mar. Drugs.

[57] Li X.-Y., Huang L.-T., Wu J.-Q., He M.-F., Zhu S.-H., Zhan P., Lv T.-F., Song Y. (2019). Zebrafish Xenograft Model of Human Lung Cancer for Evaluating Osimertinib Resistance. BioMed Res. Int..

[58] Xie X., Ross J.L., Cowell J.K., Teng Y. (2015). The promise of zebrafish as a chemical screening tool in cancer therapy. Futur. Med. Chem..

[59] Wienholds E., Schulte-Merker S., Walderich B., Plasterk R.H.A. (2002). Target-Selected Inactivation of the Zebrafish rag1 Gene. Science.

[60] Ota S., Kawahara A. (2014). Zebrafish: A model vertebrate suitable for the analysis of human genetic disorders. Congenit. Anom..

[61] Heasman J. (2002). Morpholino Oligos: Making Sense of Antisense?. Dev. Biol..

[62] Pluskota E., Dowling J.J., Gordon N., Golden J.A., Szpak D., West X.Z., Nestor C., Ma Y.-Q., Bialkowska K., Byzova T. (2011). The integrin coactivator Kindlin-2 plays a critical role in angiogenesis in mice and zebrafish. Blood.

[63] Fu C.-T., Zhu K.-Y., Mi J.-Q., Liu Y.-F., Murray S.T., Fu Y.-F., Ren C.-G., Dong Z.-W., Liu Y.-J., Dong M. (2010). An evolutionarily conserved PTEN-C/EBPα-CTNNA1 axis controls myeloid development and transformation. Blood.

[64] Wiellette E., Grinblat Y., Austen M., Hirsinger E., Amsterdam A., Walker C., Westerfield M., Sive H. (2004). Combined haploid and insertional mutation screen in the zebrafish. Genes.

[65] Haramis A.G., Hurlstone A., Van Der Velden Y., Begthel H., Born M.V.D., A Offerhaus G.J., Clevers H.C. (2006). Adenomatous polyposis coli-deficient zebrafish are susceptible to digestive tract neoplasia. EMBO Rep..

[66] Doyon Y., McCammon J.M., Miller J.C., Faraji F., Ngo C., E Katibah G., Amora R., Hocking T.D., Zhang L., Rebar E.J. (2008). Heritable targeted gene disruption in zebrafish using designed zinc-finger nucleases. Nat. Biotechnol..

[67] Amsterdam A. (2009). Zebrafish Hagoromo mutants up-regulate fgf8 postembryonically and develop neuroblastoma. Mol. Cancer Res..

[68] Lai K., Amsterdam A., Farrington S., Bronson R.T., Hopkins N., Lees J.A. (2009). Many ribosomal protein mutations are associated with growth impairment and tumor predisposition in zebrafish. Dev. Dyn..

[69] Berghmans S., Murphey R.D., Wienholds E., Neuberg D., Kutok J.L., Fletcher C.D.M., Morris J.P., Liu T.X., Schulte-Merker S., Kanki J.P. (2005). tp53 mutant zebrafish develop malignant peripheral nerve sheath tumors. Proc. Natl. Acad. Sci. USA.

[70] Langenau D.M., Feng H., Berghmans S., Kanki J.P., Kutok J.L., Look A.T. (2005). Cre/lox-regulated transgenic zebrafish model with conditional myc-induced T cell acute lymphoblastic leukemia. Proc. Natl. Acad. Sci. USA.

[71] Jung I.H., Jung D.E., Park Y.N., Song S.Y., Park S.W. (2011). Aberrant Hedgehog Ligands Induce Progressive Pancreatic Fibrosis by Paracrine Activation of Myofibroblasts and Ductular Cells in Transgenic Zebrafish. PLoS ONE.

[72] Nguyen A.T. (2012). An inducible krasV12 transgenic zebrafish model for liver tumorigenesis and chemical drug screening. Dis. Models Mech..

[73] Goessling W., North T.E., Zon L.I. (2007). New Waves of Discovery: Modeling Cancer in Zebrafish. J. Clin. Oncol..

[74] Spitsbergen J.M., Tsai H.-W., Reddy A., Miller T., Arbogast D., Hendricks J.D., Bailey G. (2000). Neoplasia in Zebrafish (*Danio rerio*) Treated with 7,12-Diniethylbenz[a]anthracene by Two Exposure Routes at Different Developmental Stages. Toxicol. Pathol..

[75] Spitsbergen J.M., Tsai H.-W., Reddy A., Miller T., Arbogast D., Hendricks J.D., Bailey G. (2000). Neoplasia in Zebrafish (*Danio rerio*) Treated with N-methyl-N′nitro-N-nitrosoguanidine by Three Exposure Routes at ifferent Developmental Stages. Toxicol. Pathol..

[76] Mizgireuv I.V., Revskoy S.Y. (2006). Transplantable Tumor Lines Generated in Clonal Zebrafish. Cancer Res..

[77] Mizgireuv I.V., Majorova I.G., Gorodinskaya V., Khudoley V.V., Revskoy S.Y. (2004). Carcinogenic Effect of N-Nitrosodimethylamine on Diploid and Triploid Zebrafish (*Danio rerio*). Toxicol. Pathol..

[78] Shepard J.L., Amatruda J.F., Finkelstein D., Ziai J., Finley K.R., Stern H.M., Chiang K., Hersey C., Barut B., Freeman J. (2007). A mutation in separase causes genome instability and increased susceptibility to epithelial cancer. Genes Dev..

[79] Vargas-Patron L.A., Agudelo-Dueñas N., Madrid-Wolff J., Venegas J.A., González J.M., Forero-Shelton M., Akle V. (2019). Xenotransplantation of Human glioblastoma in Zebrafish larvae: In vivo imaging and proliferation assessment. Biol. Open.

[80] Vittori M., Motaln H., Turnšek T.L. (2015). The Study of Glioma by Xenotransplantation in Zebrafish Early Life Stages. J. Histochem. Cytochem..

[81] Taylor A.M., Zon L.I. (2009). Zebrafish Tumor Assays: The State of Transplantation. Zebrafish.

[82] Cabezas-Sainz P., Guerra-Varela J., Carreira M.J., Mariscal J., Roel M., Rubiolo J.A., Sciara A.A., Abal M., Botana L.M., López R. (2018). Improving zebrafish embryo xenotransplantation conditions by increasing incubation temperature and establishing a proliferation index with ZFtool. BMC Cancer.

[83] Hamilton L., Astell K.R., Velikova G.V., Sieger D. (2016). A Zebrafish Live Imaging Model Reveals Differential Responses of Microglia Toward Glioblastoma Cells In Vivo. Zebrafish.

[84] Corkery D., Dellaire G., Berman J.N. (2011). Leukaemia xenotransplantation in zebrafish—Chemotherapy response assay in vivo. Br. J. Haematol..

[85] Marques I.J., Weiss F.U., Vlecken D.H., Nitsche C., Bakkers J., Lagendijk A.K., Partecke L.I., Heidecke C.-D., Lerch M.M., Bagowski C.P. (2009). Metastatic behaviour of primary human tumours in a zebrafish xenotransplantation model. BMC Cancer.

[86] Yang X.-J., Cui W., Gu A., Xu C., Yu S.-C., Li T.-T., Cui Y.-H., Zhang X., Bian X.-W. (2013). A Novel Zebrafish Xenotransplantation Model for Study of Glioma Stem Cell Invasion. PLoS ONE.

[87] Teng Y., Xie X., Walker S., White D.T., Mumm J.S., Cowell J.K. (2013). Evaluating human cancer cell metastasis in zebrafish. BMC Cancer.

[88] Augustine R., Alhussain H., Hasan A., Ahmed M.B., Yalcin H.C., Al Moustafa A.-E. (2019). A novel in ovo model to study cancer metastasis using chicken embryos and GFP expressing cancer cells. Bosn. J. Basic Med. Sci..

[89] Bentley V.L., Veinotte C.J., Corkery D., Pinder J.B., Leblanc M.A., Bedard K., Weng A.P., Berman J.N., Dellaire G. (2014). Focused chemical genomics using zebrafish xenotransplantation as a pre-clinical therapeutic platform for T-cell acute lymphoblastic leukemia. Haematologica.

[90] Ghotra V.P.S., He S., De Bont H., Van Der Ent W., Spaink H., Van De Water B., Snaar-Jagalska B.E., Danen E.H.J. (2012). Automated Whole Animal Bio-Imaging Assay for Human Cancer Dissemination. PLoS ONE.

[91] Benjamin D.C., Hynes R.O. (2017). Intravital imaging of metastasis in adult Zebrafish. BMC Cancer.

[92] Laggner C., Kokel D., Setola V., Tolia A., Lin H., Irwin J.J., Keiser M.J., Cheung C.Y.J., Minor D.L., Roth B.L. (2011). Chemical informatics and target identification in a zebrafish phenotypic screen. Nat. Chem. Biol..

[93] Terriente J., Pujades C. (2013). Use of Zebrafish Embryos for Small Molecule Screening Related to Cancer. Dev. Dyn..

[94] Ceol C.J., Houvras Y., Jane-Valbuena J., Bilodeau S., Orlando D.A., Battisti V., Fritsch L., Lin W.M., Hollmann T.J., Ferre’ F. (2011). The histone methyltransferase SETDB1 is recurrently amplified in melanoma and accelerates its onset. Nat. Cell Biol..

[95] Huiting L.N., Laroche F., Feng H. (2015). The Zebrafish as a Tool to Cancer Drug Discovery. Austin J. Pharmacol. Ther..

[96] Ridges S., Heaton W.L., Joshi D., Choi H., Eiring A., Batchelor L., Choudhry P., Manos E.J., Sofla H., Sanati A. (2012). Zebrafish screen identifies novel compound with selective toxicity against leukemia. Blood.

[97] MacRae C.A., Peterson R.T. (2003). Zebrafish-based small molecule discovery. Chem. Biol..

[98] Mbughuni M.M., Jannetto P.J., Langman L.J. (2016). Mass Spectrometry Applications for Toxicology. EJIFCC.

[99] Kiper K.G., Freeman J.L. (2019). Zebrafish as a Tool to Assess Developmental Neurotoxicity. Animal Models of Drug Addiction.

[100] Giordano G., Costa L.G. (2012). Developmental Neurotoxicity: Some Old and New Issues. ISRN Toxicol..

[101] Horzmann A.K., Freeman J. (2018). Making Waves: New Developments in Toxicology with the Zebrafish. Toxicol. Sci..

[102] Matsoukas T., Desai T., Lee K. (2015). Engineered Nanoparticles and Their Applications. J. Nanomater..

[103] Jeevanandam J., Barhoum A., Chan Y.S., Dufresne A., Danquah M.K. (2018). Review on nanoparticles and nanostructured materials: History, sources, toxicity and regulations. Beilstein J. Nanotechnol..

[104] Mostafavi E., Soltantabar P., Webster T.J. (2019). Nanotechnology and Picotechnology: A New Arena for Translational Medicine. Biomaterials in Translational Medicine.

[105] Jeevanandam J., Chan Y.S., Danquah M.K. (2019). Zebrafish as a Model Organism to Study Nanomaterial Toxicity. Emerg. Sci. J..

[106] Godin B., Touitou E. (2007). Transdermal skin delivery: Predictions for humans from in vivo, ex vivo and animal models. Adv. Drug Deliv. Rev..

[107] Saeidnia S., Manayi A., Abdollahi M. (2016). From in vitro Experiments to in vivo and Clinical Studies; Pros and Cons. Curr. Drug Discov. Technol..

[108] Cheng D., Shami G.J., Morsch M., Chung R., Braet F. (2016). Ultrastructural Mapping of the Zebrafish Gastrointestinal System as a Basis for Experimental Drug Studies. BioMed Res. Int..

[109] MacPhail R.C., Hunter D.L., Irons T.D., Padilla S. (2011). Locomotion and Behavioral Toxicity in Larval Zebrafish: Background, Methods, and Data. Zebrafish.

[110] Huang Y., Zhang J., Han X., Huang T. (2014). The Use of Zebrafish (*Danio rerio*) Behavioral Responses in Identifying Sublethal Exposures to Deltamethrin. Int. J. Environ. Res. Public Health.

[111] Bai C., Tang M. (2020). Toxicological study of metal and metal oxide nanoparticles in zebrafish. J. Appl. Toxicol..

[112] Dellinger B., Pryor W.A., Cueto R., Squadrito G.L., Hegde V., Deutsch W.A. (2001). Role of Free Radicals in the Toxicity of Airborne Fine Particulate Matter. Chem. Res. Toxicol..

[113] Li N., Sioutas C., Cho A., Schmitz D., Misra C., Sempf J., Wang M., Oberley T., Froines J., Nel A. (2003). Ultrafine particulate pollutants induce oxidative stress and mitochondrial damage. Environ. Health Perspect..

[114] Win-Shwe T.-T., Fujimaki H. (2011). Nanoparticles and Neurotoxicity. Int. J. Mol. Sci..

[115] Chakraborty C., Sarkar B.K., Hsu C.H., Wen Z.H., Lin C.S., Shieh P.C. (2009). Future prospects of nanoparticles on brain targeted drug delivery. J. Neuro-Oncol..

[116] Li X. (2014). SiO_2_ nanoparticles change colour preference and cause Parkinson’s-like behaviour in zebrafish. Sci. Rep..

[117] Zhang W., Sun X., Chen L., Lin K.-F., Dong Q.-X., Huang C.-J., Fu R.-B., Zhu J. (2012). Toxicological effect of joint cadmium selenium quantum dots and copper ion exposure on zebrafish. Environ. Toxicol. Chem..

[118] Sheng L. (2016). Mechanism of TiO_2_ nanoparticle-induced neurotoxicity in zebrafish (*Danio rerio*). Environ. Toxicol..

[119] Parng C., Roy N.M., Ton C., Lin Y., McGrath P. (2007). Neurotoxicity assessment using zebrafish. J. Pharmacol. Toxicol. Methods.

[120] Powers C.M., Slotkin T.A., Seidler F.J., Badireddy A.R., Padilla S. (2011). Silver nanoparticles alter zebrafish development and larval behavior: Distinct roles for particle size, coating and composition. Neurotoxicol. Teratol..

[121] de Oliveira G.M.T. (2014). Transient modulation of acetylcholinesterase activity caused by exposure to dextran-coated iron oxide nanoparticles in brain of adult zebrafish. Comp. Biochem. Physiol. Part C Toxicol. Pharmacol..

[122] Zakaria Z.Z., Benslimane F., Nasrallah G., Shurbaji S., Younes N.N., Mraiche F., Da’As S.I., Yalcin H.C. (2018). Using Zebrafish for Investigating the Molecular Mechanisms of Drug-Induced Cardiotoxicity. BioMed Res. Int..

[123] Benslimane F., Zakaria Z.Z., Shurbaji S., Abdelrasool M.K.A., Al-Badr M.A.H., Al Absi E.S.K., Yalcin H.C. (2020). Cardiac function and blood flow hemodynamics assessment of zebrafish (*Danio rerio*) using high-speed video microscopy. Micron.

[124] Yalcin H.C. (2018). Hemodynamic Studies for Analyzing the Teratogenic Effects of Drugs in the Zebrafish Embryo. Advanced Structural Safety Studies.

[125] Benslimane F., Alser M., Zakaria Z.Z., Sharma A., Abdelrahman H.A., Yalcin H.C. (2019). Adaptation of a Mice Doppler Echocardiography Platform to Measure Cardiac Flow Velocities for Embryonic Chicken and Adult Zebrafish. Front. Bioeng. Biotechnol..

[126] Da’As S.I., Yalcin H.C., Nasrallah G.K., Mohamed I.A., Nomikos M., Yacoub M.H., Fakhro K.A. (2020). Functional characterization of human myosin-binding protein C3 variants associated with hypertrophic cardiomyopathy reveals exon-specific cardiac phenotypes in zebrafish model. J. Cell. Physiol..

[127] Yalcin H.C., Amindari A., Butcher J., Althani A., Yacoub M. (2017). Heart function and hemodynamic analysis for zebrafish embryos. Dev. Dyn..

[128] Salman H.E., Yalcin H.C. (2020). Advanced blood flow assessment in Zebrafish via experimental digital particle image velocimetry and computational fluid dynamics modeling. Micron.

[129] Chang J., Ichihara G., Shimada Y., Tada-Oikawa S., Kuroyanagi J., Zhang B., Suzuki Y., Sehsah R., Kato M., Tanaka T. (2015). Copper Oxide Nanoparticles Reduce Vasculogenesis in Transgenic Zebrafish Through Down-Regulation of Vascular Endothelial Growth Factor Expression and Induction of Apoptosis. J. Nanosci. Nanotechnol..

[130] Cui B., Ren L., Xu Q.-H., Yin L.-Y., Zhou X.-Y., Liu J.-X. (2016). Silver_ nanoparticles inhibited erythrogenesis during zebrafish embryogenesis. Aquat. Toxicol..

[131] Villacis R.A., Filho J.S., Piña B., Azevedo R.B., Pic-Taylor A., Mazzeu J., Grisolia C.K. (2017). Integrated assessment of toxic effects of maghemite (γ-Fe_2_O_3_) nanoparticles in zebrafish. Aquat. Toxicol..

[132] Li Y., Sun Y., Zhang G., He Z., Wang Y., Cui J. (2016). Effects of copper oxide nanoparticles on developing zebrafish embryos and larvae. Int. J. Nanomed..

[133] Yu L.-P., Fang T., Xiong D.-W., Zhu W.-T., Sima X.-F. (2011). Comparative toxicity of nano-ZnO and bulk ZnO suspensions to zebrafish and the effects of sedimentation, ˙OH production and particle dissolution in distilled water. J. Environ. Monit..

[134] Qin X., Laroche F.F.J., Peerzade S.A.M.A., Lam A., Sokolov I., Feng H. (2020). In Vivo Targeting of Xenografted Human Cancer Cells with Functionalized Fluorescent Silica Nanoparticles in Zebrafish. J. Vis. Exp..

[135] Singh M., Murriel C.L., Johnson L. (2012). Genetically Engineered Mouse Models: Closing the Gap between Preclinical Data and Trial Outcomes: Figure 1. Cancer Res..

[136] Sharkey E.F., Fogh J. (1984). Considerations in the use of nude mice for cancer research. Cancer Metastasis Rev..

[137] Dadwal A., Baldi A., Narang R.K. (2018). Nanoparticles as carriers for drug delivery in cancer. Artif. Cells Nanomed. Biotechnol..

[138] Senapati S., Mahanta A.K., Kumar S., Maiti P. (2018). Controlled drug delivery vehicles for cancer treatment and their performance. Signal Transduct. Target. Ther..

[139] Huff T.B., Tong L., Zhao Y., Hansen M.N., Cheng J.-X., Wei A. (2007). Hyperthermic effects of gold nanorods on tumor cells. Nanomed..

[140] Gao J. (2007). FePt@ CoS2 yolk− shell nanocrystals as a potent agent to kill HeLa cells. J. Am. Chem. Soc..

[141] Asharani P.V., Lianwu Y., Gong Z., Valiyaveettil S. (2010). Comparison of the toxicity of silver, gold and platinum nanoparticles in developing zebrafish embryos. Nanotoxicology.

[142] Balogh L. (2002). Dendrimer nanocompositer in medicine. Chim. Oggi.

[143] Bodewein L., Schmelter F., Di Fiore S., Hollert H., Fischer R., Fenske M. (2016). Differences in toxicity of anionic and cationic PAMAM and PPI dendrimers in zebrafish embryos and cancer cell lines. Toxicol. Appl. Pharmacol..

[144] Wu S.-Y., Chou H.-Y., Tsai H.-C., Anbazhagan R., Yuh C.-H., Yang J.M., Chang Y.-H. (2020). Amino acid-modified PAMAM dendritic nanocarriers as effective chemotherapeutic drug vehicles in cancer treatment: A study using zebrafish as a cancer model. RSC Adv..

[145] Kocere A., Resseguier J., Wohlmann J., Skjeldal F.M., Khan S., Speth M., Dal N.-J.K., Ng M.Y.W., Alonso-Rodriguez N., Scarpa E. (2020). Real-time imaging of polymersome nanoparticles in zebrafish embryos engrafted with melanoma cancer cells: Localization, toxicity and treatment analysis. EBioMedicine.

[146] Croissant J.G., Cattoën X., Durand J.-O., Man M.W.C., Khashab N.M. (2016). Organosilica hybrid nanomaterials with a high organic content: Syntheses and applications of silsesquioxanes. Nanoscale.

[147] Dib S., Aggad D., Jimenez C.M., Lakrafi A., Hery G., Nguyen C., Durand D., Morère A., El Cheikh K., Sol V. (2019). Porphyrin-based bridged silsesquioxane nanoparticles for targeted two-photon photodynamic therapy of zebrafish xenografted with human tumor. Cancer Rep..

[148] Nadar R.A., Asokan N., Degli Esposti L., Curci A., Barbanente A., Schlatt L., Karst U., Iafisco M., Margiotta N., Brand M. (2020). Preclinical evaluation of platinum-loaded hydroxyapatite nanoparticles in an embryonic zebrafish xenograft model. Nanoscale.

[149] Xu Z., Zhang Y.-L., Song C., Wu L.-L., Gao H.-W. (2012). Interactions of Hydroxyapatite with Proteins and Its Toxicological Effect to Zebrafish Embryos Development. PLoS ONE.

[150] Peerzade S.A.M.A., Qin X., Laroche F.J.F., Palantavida S., Dokukin M., Peng B., Feng H., Sokolov I. (2019). Ultrabright fluorescent silica nanoparticles for in vivo targeting of xenografted human tumors and cancer cells in zebrafish. Nanoscale.

[151] Duan J., Yu Y., Shi H., Tian L., Guo C., Huang P., Zhou X., Peng S., Sun Z. (2013). Toxic Effects of Silica Nanoparticles on Zebrafish Embryos and Larvae. PLoS ONE.

